# Generalisation of Social Communication Skills by Autistic Children During Play-Based Assessments Across Home, School and an Unfamiliar Research Setting

**DOI:** 10.1007/s10803-024-06370-x

**Published:** 2024-05-14

**Authors:** Sophie Carruthers, Tony Charman, Kathy Leadbitter, Ceri Ellis, Lauren Taylor, Heather Moore, Carol Taylor, Kirsty James, Matea Balabanovska, Sophie Langhorne, Catherine Aldred, Vicky Slonims, Vicki Grahame, Patricia Howlin, Helen McConachie, Jeremy Parr, Richard Emsley, Ann Le Couteur, Jonathan Green, Andrew Pickles, and the PACT-G Trial Group

**Affiliations:** 1 Department of Psychology, Institute of Psychiatry, Psychology and Neuroscience, King’s College London, London, UK; 2 Division of Psychology and Mental Health, University of Manchester, Manchester, UK; 3 Population Health Sciences Institute, Newcastle University, Sir James Spence Institute, Royal Victoria Infirmary, Newcastle Upon Tyne, UK; 4 Department of Biostatistics & Health Informatics, Institute of Psychiatry, Psychology & Neuroscience, King’s College London, London, UK; 5 Evelina London Children’s Hospital, Guy’s & St Thomas’ NHS Foundations Trust, London, UK; 6 Department of Child & Adolescent Psychiatry, Institute of Psychiatry, Psychology and Neuroscience, King’s College London, London, UK; 7 Complex Neurodevelopmental Disorders Service (CNDS), Cumbria, Northumberland, Tyne and Wear NHS Foundation Trust, London, UK; 8 Great North Children’s Hospital, Newcastle Upon Tyne NHS Foundation Trust, London, UK; 9 Manchester Academic Health Sciences Centre, Manchester Royal Children’s Hospital, University of Manchester, Manchester, UK

**Keywords:** Autism, Development, Social communication, Generalisation

## Abstract

We investigated autistic children’s generalisation of social communication over time across three settings during a play-based assessment with different adults and explore the potential moderating effects on generalisation of age, nonverbal IQ and level of restricted and repetitive behaviours. The social communication abilities of 248 autistic children (2–11 years, 21% female, 22% single parent, 60% white) from three UK sites were assessed from 1984 video interactions in three contexts with three different interaction partners (parent/home, teaching assistant/school, researcher/clinic) at baseline, midpoint (+ 7m) and endpoint (+ 12m) within the Paediatric Autism Communication Trial-Generalised (PACT-G), a parent-mediated social communication intervention. Children’s midpoint social communication at home generalised to school at midpoint and to clinic at endpoint. Generalisation was stronger from home to school and clinic than school to home and clinic. Generalisation was not moderated by age, nonverbal IQ or restricted and repetitive behaviour. Broader child development did not explain the pattern of results. The current study is the largest study to date to explore generalisation with autistic children and provides novel insight into their generalisation of social communication skills. Further research is needed to gain a more comprehensive understanding of facilitators of generalisation across settings and interaction partners in order to develop targeted strategies for interventions to enhance outcomes for young autistic children.

**Supplementary Information:**

The online version contains supplementary material available at 10.1007/s10803-024-06370-x.

## Generalisation in Early Autism Intervention Studies

Demonstrating the transmission of targeted proximal intervention effects into broader, generalisable functional change in everyday contexts is a key current challenge for early autism intervention research (Carruthers et al., 2020; Crank et al., 2021; Fuller & Kaiser, 2020; Sandbank et al., 2021). In the past decade multiple studies have demonstrated that intervention effects sizes for naturalistic developmental behavioural interventions are smaller for distal vs. proximal and for context-bound vs. more generalisable outcomes (Crank et al., 2021; Hong et al., 2018; Sandbank et al., 2021, 2023; Yoder et al., 2013). The capacity to generalise acquired skills flexibly across different situations, people and contexts—a central feature of skill acquisition in neurotypical development—has often been suggested to be a core difficulty for autistic children. However, the robustness of the evidence for this view and our understanding of the underlying processes that lead to limited generalisation remain uncertain (Carruthers et al., 2020; Stokes & Osnes, 2016; Swan et al., 2016). A systematic review of randomised controlled trials of early social communication interventions for autistic children showed that eight out of nine trials reviewed found some successful generalisation of skills across people and/or setting (Carruthers et al., 2020).

Factors associated with intervention strategies, such as over-prompting and artificial learning contexts dissociated from real-world social-pragmatic contexts, may create barriers to generalisation of child skills. One plausible approach to overcome some of these difficulties is to embed interventions into real-world social environments through parent- and teacher-mediated learning, which may optimise the interpersonal cues and continuity of learning across contexts (Schreibman et al., 2015). This increases opportunities for incidental or naturalistic teaching by weaving functional social and communication learning into the child’s daily experiences where these skills are required. Although most psychosocial interventions target specific behaviours and skills, the underlying models work on the assumption that there will be a generalised developmental shift consequent on initial changes in the targeted domains. Developmentally-informed interventions build on theories of neurotypical development of the social mind (Carpendale & Lewis, 2004; Johnson, 2011) in which there are progressive cascades of impacts across a broadening range of behavioural domains and settings (Green, 2022; Green & Garg, 2018).

## Mediators and Moderators of Generalised Outcomes

Few parent-mediated autism intervention trials have specifically examined the downstream effect of change in parenting behaviours (i.e. the focus of the intervention strategy) on generalised child social communication skills. One study demonstrated that change in parenting behaviour mediated the effect on the initial intervention target (child initiations within parent–child communication) and that child initiations, in turn, mediated the effect in the generalised social communication context with an unfamiliar interaction partner during the Autism Diagnostic Observation Schedule (ADOS; Pickles et al., 2015). Follow-up of the same cohort six years later demonstrated that a sustained increase in this child initiation with their parent continued to mediate the treatment effect on ADOS scores six years after the intervention period (Carruthers et al., 2023). An educator-mediated social communication intervention trial also demonstrated a downstream generalisation cascade in which child-initiated joint engagement was related to joint attention initiations, which in turn led to improvements in language outcome (Shih et al., 2021). Few other intervention trials have explored possible moderators of generalisation effects of any skill type among autistic children. However, in relation to intervention outcomes in general, age and IQ have been proposed as moderators of intervention effect, although the evidence for this is mixed (Klinger et al., 2021). More broadly, for autistic individuals, the flexibility required to adapt behaviour to different social exchanges may be lower for individuals with higher levels of restricted and repetitive behaviour (D’Cruz et al., 2013).

## A Framework for Testing Generalisation Across Time, Context and People

Whilst interventions are one example of a learning environment, most children’s learning comes from the family and school environments within which they spend most time. However, research exploring the relative contribution and dynamics of these two core learning environments for autistic children is rare. If we understood better how different learning environments interact, we could tailor interventions more precisely to support naturally occurring learning dynamics across home and school contexts. In order to advance our understanding of generalisation of social communication across different settings and partners we need observational capture of social exchanges across contexts that offer similar expectations and opportunities rated by a consistent coding framework. Until recently, the autism field did not have an instrument that was sufficiently flexible to be used as an observational measure of social communication across different settings and people. The Brief Observation of Social Communication Change (Grzadzinski et al., 2016) is a relatively new, play-based assessment, designed to measure changes in social communication skills over the course of interventions. Frost and colleagues (Frost et al., 2019) tested whether the BOSCC could be used to assess similarities and differences in social communication when a child was interacting with their parent during a play context and a snack context. The Social Communication subscale exhibited strong psychometric properties and indicated that similar information was captured across the different contexts. In contrast, the second subscale, the Restricted and Repetitive Behaviour (RRB) subscale was less consistent in performance, with weaker psychometric properties and a significantly different profile of scores across the two settings. Other studies have also reported weaker psychometric properties for the RRB subscale (Carruthers et al., 2021; Kim et al., 2019).

## The Current Study: Testing Generalisation in the Context of the PACT-G Trial

The fact that the BOSCC can be used with different adult interaction partners across different settings was central to its use as a secondary outcome measure in the Paediatric Autism Communication Trial-Generalised (PACT-G; Green et al., 2022). Autistic children (aged 2–11) were assessed with BOSCC on their social communication skills with a parent at home, teaching assistant at school and a researcher in a university environment at multiple time points over the 12-month trial duration. The PACT-G trial did not demonstrate a greater effect than treatment-as-usual on measures of autism symptoms (including the BOSCC), language or social adaptation. However, it did result in greater improvement in children’s dyadic social communicative initiations during interactions with the parent and teaching assistant. Mediation analysis showed that the increase in children’s communicative initiations in both home and education settings was a consequence of the adjusted communication styles of parents and teaching staff, respectively (Green et al., 2022).

Whilst no overall intervention effect was demonstrated on the BOSCC, the longitudinal design of the trial offered an opportunity to investigate, across the whole trial cohort, a novel analysis of the comparison of acquired skills in autism development across time, context and person. The current study, therefore, is an investigation of overall developmental processes in the PACT-G cohort, not an investigation of treatment effects or their mediation. We aimed to investigate children’s generalisation of social communication over time during a play-based assessment with three different adults in three different settings, and to explore the potential moderating effects on generalisation of age, nonverbal IQ and level of restricted and repetitive behaviours.

## Methods

### Participants

Two hundred and forty-eight (197 males, 51 female) children, aged between 2 and 11 years, participated in PACT-G (Green et al., 2022). PACT-G was a three-site, two-group, randomised controlled trial of the PACT-G social communication intervention plus treatment as usual (TAU) compared to TAU alone (ISRCTN Registration: 25378536). Children included in the trial had clinical diagnoses of autism, confirmed using the Autism Diagnostic Observation Schedule-2nd Edition (Lord et al., 2012) and Social Communication Questionnaire (Rutter et al., 2003) at trial baseline. Children were recruited from local clinical and educational services. All children had nonverbal age equivalent scores of more than 12 months as measured by the Mullen Scales of Early Learning and those aged 5 years and older were between P3 (beginning to use ‘intentional communication) and P8 (a language age equivalent of up to 4 years) on an English National Curriculum assessment. Parents were required to have sufficient English to participate in the PACT-G assessments and intervention and needed to report speaking to their child at home in English at least some of the time. Neither children nor parents had any known severe hearing or visual impairments; parents had no significant psychiatric conditions or learning disabilities. Exclusion criteria included a sibling already in the trial, participation in the pilot phase of the trial, a child having a non-verbal age-equivalent level of ≤ 12 months, epilepsy not controlled by medication, any safeguarding concerns or family situation that would affect participation in the trial, or any child with an identified genetic disorder that would impact on ability to participate or affect validity of the data. For a family to participate, the child’s school also had to sign up to the study. Child and family characteristics are presented in Table 1.

**Table 1:** Summary of baseline participant characteristics by intervention group

	TAU *M *(SD) (*n* = 127)	PACT-G *M *(SD) (*n* = 121)
Age (years) preschool (*n* = 151)	3.9 (0.7)	4.1 (0.6)
Age (years) school-age (*n* = 97)	6.9 (1.4)	7.4 (1.6)
Female	27/127 (21%)	24/121 (20%)
Ethnicity		
White	73/127 (57%)	76/121 (63%)
Black	21/127 (17%)	19/121 (16%)
Asian	16/127 (13%)	13/121 (11%)
Mixed	17/127 (13%)	6/121 (5%)
Other	0/121 (0%)	7/121 (6%)
No second parent in household	28/127 (22%)	27/121 (22%)
Languages spoken		
English only	98/127 (77%)	99/121 (82%)
Other only	3/127 (2%)	1/121 (1%)
English and other	26/127 (20%)	21/121 (17%)
ADOS-2		
Module 1	*n* = 95	*n* = 92
Module 2	*n* = 32	*n* = 29
ADOS-2 total		
Module 1	19.7 (3.51)	19.5 (3.10)
Module 2	15.1 (3.50)	16.4 (3.89)
RBQ		
Module 1	*n* = 90	*n* = 89
Module 2	*n* = 29	*n* = 27
RBQ-Sensory Motor		
Module 1	10.7 (5.16)	9.94 (4.77)
Module 2	11.4 (5.84)	7.44 (4.14)
RBQ-Insistence on Sameness		
Module 1	7.47 (5.59)	7.77 (5.68)
Module 2	12.3 (7.46)	10.2 (6.18)
Non-verbal IQ age equivalent (months)		
Module 1	24.04 (7.12)	24.74 (7.75)
Module 2	38.5 (15.1)	37.0 (9.59)
MSEL Non-verbal IQ (VR T-score)	25.2 (11.7)	22.9 (8.0)
MSEL Non-verbal IQ age equivalent (months) below median (range 13–24)	*n* = 63/127	*n* = 52/121
MSEL Non-verbal DQ	48.8 (22.2)	46.5 (17.7)
Language scores	*n* = 57	*n* = 46
Receptive one-word	29.4 (22.5)	32.1 (19.3)
Receptive one-word (raw score)	*n* = 62	*n* = 58
Expressive one-word	28.2 (19.2)	32.0 (12.9)
Expressive one-word (raw score)		
MacArthur CDI	*n* = 120	*n* = 119
MCDI words understood	202.4 (128.4)	213.4 (134.7)
MCDI words understood and said	139.1 (141.5)	152.1 (144.3)

*TAU* Treatment as Usual*, Module 1 and Module 2* Modules on the ADOS-2, *ADOS-2* Autism Diagnostic Observation Schedule (2nd version), *RBQ* Repetitive Behaviour Questionnaire, *MSEL* Mullen Scales of Early Learning, *VR* Visual Reception, *DQ* Developmental Quotient (Age Equivalent/Chronological Age × 100)

### Ethical Considerations

All procedures involving human participants were in accordance with the ethical standards of the North West-Greater Manchester Central Research Ethics Committee (REF: 15/NW/0912). Parents provided informed, written consent before participating in PACT-G. In addition, a delegated representative from each school signed a Memorandum of Agreement prior to their school’s participation in PACT-G. Further details are outlined in Green et al. (2022).

### PACT-G Trial

The PACT-G trial ran in London, Greater Manchester and the North-East of England. Randomisation allocation was stratified by intervention site, age strata (< 5 years, 5 years and older), and gender. Assessments took place at baseline, midpoint (+ 7 months) and endpoint (+ 12 months).

PACT-G therapy was an adaptation of the original clinic-based PACT therapy (Green et al., 2010) into a multicomponent intervention delivered simultaneously in home and school. The rationale of PACT is that synchronous, sensitive and responsive communication from the adult increases dyadic communication and social interaction skills in the child, which then may generalise into functioning in other contexts. This logic model is supported by mediation analysis from two clinic-based PACT trials (Aldred et al., 2012; Pickles et al., 2015), which demonstrated that improved parent synchrony was associated with increased child dyadic initiation, which in turn was associated with better generalisation of social communication in a research evaluation context. PACT-G aimed to build on this evidence by implementing features designed to further support the generalisation of the child’s learning across contexts. For instance, the therapy took place within the naturalistic settings of both home and school with the child present, integrated the parental techniques into daily routines and play, and included teaching assistants as direct recipients of the therapy in parallel to parents. Parents and teaching assistants were supported, using video feedback, to interact with the child using evidence-based strategies that facilitate social communication development in the child. Further details of the clinic-based PACT intervention can be found the supplementary materials of Green et al. (2010). Description of the modifications for PACT-G are reported in (Green et al., 2022).

### Measures

#### Brief Observation of Social Communication Change (BOSCC)

The BOSCC (Grzadzinski et al., 2016) is an outcome tool designed to measure changes in children’s social communication skills that can be used across different people and settings. Behaviours are scored from a naturalistic adult–child play-based exchange across items with a six-point scale. In PACT-G, the BOSCC assessed each child’s social communication during play with a parent at home, a teaching assistant at school, and a researcher in the research setting. Home and school BOSCC assessments took place at baseline, midpoint and endpoint, whilst research BOSCCs occurred only at baseline and endpoint. For PACT-G, Module 1 (75.4% of participants; Version July 27, 2017) and Module 2 (24.6% of participants; 2019 version) were used, consisting of 15 or 20 items, respectively. The core items make up two subscales: social communication and restricted and repetitive behaviour. In the current analysis, only the social communication items are used, comprising 8 items in Module 1 (maximum score 40) and 12 items in Module 2 (maximum score 60). Across Module 1 and 2, BOSCC coding was conducted on two video-recorded play segments, each made up of 4 min of free play and 1 min of bubble play. Further details on the administration, including the social communication behaviours rated for Module 1 and Module 2, can be found in Supplementary Materials. As part of the trial design the BOSCC Module was kept consistent across timepoints so that children did not change from BOSCC Module 1 to Module 2 across the study.

For each child, there were eight BOSCC assessments: three at school, three at home (each baseline, midpoint and endpoint) and two with the researcher (baseline and endpoint), totalling 1,984 assessments. The Module 1 BOSCCs were coded by 14 individuals based in the UK and the US, whilst all Module 2 BOSCCs were coded by 6 US-based individuals from the team developing the measure. All coders were blind to group and timepoint. A random sub-sample of BOSCCs, stratified by rater, were multiple coded for formal reliability analyses (63 Module 1 and 48 Module 2 tapes). Intra-class correlations were calculated to be 0.87 (95% confidence interval [CI] 0.81, 0.91) for module 1, 0.86 (CI 0.76, 0.92) for module 2, and overall 0.86 (CI 0.76, 0.92).

#### Mullen Scales of Early Learning (MSEL)

The baseline visual reception age equivalent of the MSEL (Mullen, 1995) was used to explore the moderating effect of non-verbal IQ. Given the overall low DQ of the sample (see Table 1) a median split was used to transform the scores into a binary variable dividing the sample into two near-equal size groups (age equivalent 13 to 24 months vs. 25 to 69 months).

#### Repetitive Behaviour Questionnaire (RBQ)

The RBQ (Honey et al., 2012) is a parent-report measure of repetitive and restricted behaviours, administered at baseline. The two subdomains of Insistence on Sameness and Sensory Motor Behaviours were used independently to explore the moderating effect of restricted and repetitive behaviour levels.

### Data Analysis

The analyses presented were preregistered: https://aspredicted.org/blind.php?x=dw43ak. Minor alterations between the preregistration and final analyses are outlined in Supplementary Materials. While the current study set out to explore generalisation using both subscales of the BOSCC, the length and play-based setting was considered to elicit too few RRBs to analyse these reliably as a distinct construct. The analysis reported therefore focuses on only the social communication subscale.

Pearson correlations were calculated between context and timepoint. Correlations are interpreted using *r* of $\ge$0.1 representing a small effect size (ES), $\ge$ 0.3 a medium ES and $\ge$ 0.5 a large ES (Cohen, 1988).

**Fig. 1:**
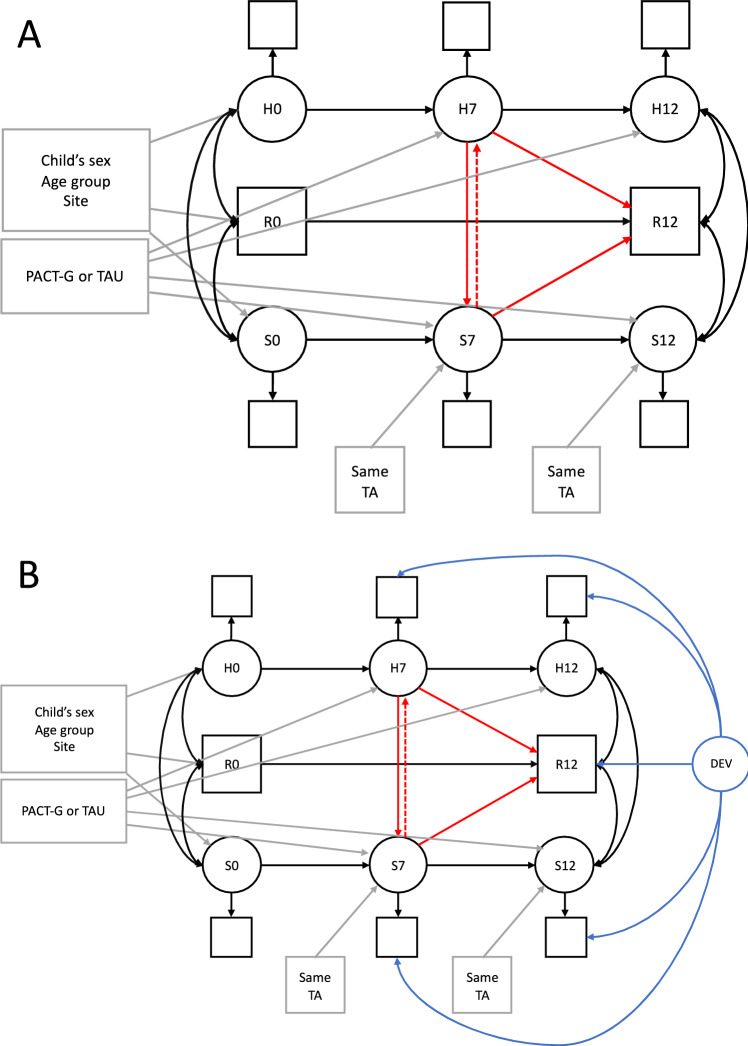
Structural equation models fitted to baseline (0 m), midpoint (7 m) and endpoint (12 m) BOSCC data from home, school and research settings. **A** represents the initial model tested. **B** represents the second model tested with the addition of a latent variable representing broader (unmeasured) child development. *DEV* development, *H* home, *R* research, *S* school. Numerical suffix = month from start of therapy. Circles represent latent variables; squares represent observed variables. Grey arrows represent the paths accounting for baseline covariates and whether the child was in the PACT-G intervention or TAU group. Red errors represent generalisation paths. **B** the blue lines represent the loadings for the latent factor representing child development. Covariation paths are double headed curved paths. *PACT-G* Paediatric Autism Communication Trial-Generalised, *TA* teaching assistant, *TAU* treatment as usual

Generalisation was examined by fitting models using Mplus (Muthén & Muthén, 2017) of the form shown in Fig. 1. The model was stratified by BOSCC module as a two-group KNOWNCLASS model and estimated by maximum likelihood. All available measurements were included in the analysis under an assumption of a missing at random missing data mechanism. Goodness of fit was evaluated with RMSEA and CFI, where satisfactory fit is indicated by values below 0.08 and above 0.90, respectively (Kline, 2015). The results of Wald tests used to confirm constraints are reported in Table S1 (See Supplementary Material). Before the three-context model, models were run for two contexts in order to explore results individually for the (i) home and research and (ii) school and research settings. Unless otherwise specified, parameters were unconstrained across module.

#### Measurement Model

The three repeated measurements of home and school BOSCC assessments permitted measurement error to be accounted for through the use of latent variables, as has been done with previous studies (Carruthers et al., 2023; Pickles et al., 2015). Loadings of each latent variable onto the respective observed variable were fixed to 1 and, within setting, residual variances were constrained to be equal across timepoints and BOSCC module. As the researcher-BOSCC only had two assessment timepoints (and therefore insufficient to account for the measurement error using a simplex model), these remained as observed variables in the model. As the BOSCC ratings were made blind from entirely independently recorded video, the model did not include correlated errors, either within-contexts over time or across-contexts.

#### Structural Model

##### Within-Context Observations

First-order autoregressive paths are added within the home, school or research contexts, unconstrained over time and Module.

##### Between-Context Covariances

Covariances across BOSCC contexts at baseline and endpoint were unconstrained over Module, as were all intercepts.

##### Trial Design

Unconstrained paths are placed between the dummy variables representing the stratifiers (child’s age group, child’s sex and intervention site) and the three baseline BOSCC measurements. Since therapists did not work directly with the child and intervention was assigned at random, no paths were included from the intervention variable to the baseline BOSCC measurements and, for post-baseline measures, only to school and home measurements. Paths from intervention group to school BOSCC and from intervention group to home BOSCC are constrained equal across Module.

##### Change in School Staff Partner

Changes in the school-based partner during the trial were modelled with paths from dummy variables to the school context BOSCC assessments to allow for a mean difference where a change in teaching assistant had occurred, constrained equal across Module and timepoint.

##### Cross-Lagged Cross-Context (Generalisation) Paths

We began with bivariate models testing separately home-researcher and school-researcher context pairs. However, as the generalisation arising from the omitted play partner may be considered an omitted confounder in these bivariate models, a further model was developed including all three contexts. These models included reciprocal home-school paths between midpoint factors which were explored as part of the analysis. Following previous experience (Goldsmith et al., 2018) that contemporaneous effects tend to be stronger than lagged ones, the home-school/school-home paths between midpoint and endpoint were left unexplored.

To explore generalisation across contexts, key paths were added between contexts, namely (1) home midpoint to school midpoint, (2) home midpoint to researcher endpoint, and (3) school midpoint to researcher endpoint. To maximize power, these unstandardised generalisation path coefficients were constrained equal across Module, though standardised coefficients would not be expected equal.

#### Model Including Broader Development

To explore the potentially confounding role of unmeasured development, a second model shown in Fig. 1B was tested that included a latent factor representing unmeasured child development over the duration of the trial.

#### Moderating Factors

We had pre-specified baseline age, nonverbal IQ and level of restrictive and repetitive behaviours and interests as potential moderators. We chose nonverbal IQ (Visual Reception score on the Mullen) as a moderator in our analysis for both pragmatic and theoretical reasons. Pragmatically, we administered only the nonverbal subscales of the Mullen at baseline (as per Green et al., 2022) and considered the Visual Reception subscale the best measure of nonverbal ‘cognitive potential’ for the current sample of children. Theoretically, language abilities are most strongly associated with BOSCC social communication scores (Grzadzinski et al., 2016). Correlations among other key behavioural indicators (Table S2—See Supplementary Material) suggested no additional candidates. We tested whether each of the three factors moderated the three generalisation paths using the XWITH command and each moderating variable standardised across groups (Klein & Moosbrugger, 2000; Maslowsky et al., 2015). The main and interaction effects of the moderating variable were constrained equal across Modules. The models were estimated via maximum likelihood, with standard error approximation using the first-order derivative (MLF).

## Results

Missing BOSCC data points across all available time points were 5.2% for the researcher setting, 8.7% for the home setting and 8.1% for the school setting. Mean scores, change scores and standard deviations are reported in Table 2. Correlations between each timepoint and context are reported in Table 3. All correlations were large, ranging between 0.44 and 0.65. Our modelling examines how these measures might be influencing each other over time across the whole cohort, and thus though we adjust for possible intervention effects, we are not concerned here with actual treatment effects, mediated or otherwise.

**Table 2:** BOSCC social communication subscale by timepoint, context and ADOS module

	Baseline *M* (SD)	Midpoint *M* (SD)	Endpoint *M* (SD)
Research Module 1	30.9 (5.62) *n* = 186		28.3 (7.27) *n* = 180
Research Module 2	36.3 (10.9) *n* = 51		31.6 (13.0) *n* = 53
Home Module 1	27.9 (6.41) *n* = 182	27.1 (7.26) *n* = 164	26.5 (7.67) *n* = 172
Home Module 2	31.6 (11.1) *n* = 54	28.6 (10.6) *n* = 52	27.1 (10.6) *n* = 55
School Module 1	29.4 (6.93) *n* = 183	27.4 (7.17) n = 172	27.3 (8.39) *n* = 170
School Module 2	33.0 (10.7) *n* = 54	30.4 (12.8) *n* = 53	30.5 (12.6) *n* = 52

Higher BOSCC scores indicate higher levels of autistic characteristics

**Table 3:** Pearson correlations between the BOSCC assessment timepoints and contexts

	Research baseline	Research endpoint	Home baseline	Home midpoint	Home endpoint	School baseline	School midpoint
Research endpoint	0.57						
Home baseline	0.48	0.48					
Home midpoint	0.54	0.65	0.58				
Home endpoint	0.48	0.57	0.53	0.65			
School baseline	0.58	0.47	0.50	0.54	0.51		
School midpoint	0.56	0.51	0.55	0.65	0.56	0.58	
School endpoint	0.47	0.61	0.50	0.62	0.56	0.44	0.60

All correlations were *p* < .001 with Bonferroni correction

### Cross Lagged Analysis: Social Communication Subscale

#### Initial Two-Context Models

First, we ran the model using only BOSCC social communication data from parent and researcher assessments. Model fit was good with RMSEA = 0.059 and CFI = 0.964. The path from home midpoint to researcher endpoint BOSCC score, constrained equal across Module, was significant (*p* < 0.001) with a standardised coefficient of 0.72 (95% CI 0.55, 0.88) for Module 1 and 0.68 (0.51, 0.85) for Module 2.

Second, we ran a similar two-context model for the teaching assistant (TA) and researcher assessments, where the model fit was also good with RMSEA = 0.031 and CFI = 0.985. The path from TA midpoint to endpoint researcher was significant (*p* < 0.001) with standardised coefficients of 0.41 (0.26, 0.56) for Module 1 and 0.42 (0.26, 0.58) for Module 2. Eighty-five (34.3%) children experienced a change in TA between baseline and midpoint, and 94 (37.9%) children between midpoint and endpoint. Twenty-two children (8.9%) experienced changes at both timepoints. Therefore, 157 (63.3%) children experienced at least one TA change. However, changing TA did not significantly alter the expected level of BOSCC-coded autistic behaviour.

#### Three Context Model

For the model spanning all three contexts, the model fit was good with RMSEA = 0.023 and CFI = 0.992. Direct paths from home midpoint to researcher endpoint, home midpoint to school midpoint, and school midpoint to researcher endpoint are given in Table 4. In addition to the direct path between home midpoint and researcher endpoint, there is an indirect path between the two via school midpoint. The total path from home midpoint to researcher endpoint is also provided in Table 4, which incorporates the direct and indirect path.

**Table 4:** Results of structural equation model for BOSCC social communication subscale across assessment with parent, teaching assistant and researcher (unstandardized effects constrained across module)

	Model 1 without development factor			Model 2 with development factor		
	Standardised Coefficient	95% Confidence Intervals	*p* value	Standardised Coefficient	95% Confidence Intervals	*p* value
Direct paths						
Parent midpoint to teaching assistant midpoint						
Module 1^†^	0.99	0.63, 1.36	< .001	0.99	0.62, 1.36	< .001
Module 2^†^	0.75	0.56, 0.95	< .001	0.75	0.55, 0.96	< .001
Parent midpoint to researcher endpoint						
Module 1^†^	0.85	0.46, 1.24	< .001	0.85	0.46, 1.24	< .001
Module 2^†^	0.81	0.38, 1.24	< .001	0.81	0.38, 1.24	< .001
Teaching assistant midpoint to researcher endpoint						
Module 1^†^	− .20	− 0.57, 0.17	0.285	− 0.20	− 0.57, 0.17	0.283
Module 2^†^	− .25	− 0.71, 0.21	0.285	− 0.25	− 0.71, 0.21	0.283
Indirect paths						
Parent midpoint to researcher endpoint (via* teaching assistant midpoint)*						
Module 1	− 0.20	− 0.56, 0.17	0.285	− 0.20	− 0.56, 0.16	0.283
Module 2	− 0.19	− 0.55, 0.17	0.301	− 0.19	− 0.55, 0.17	0.299
Total paths						
Parent midpoint to researcher endpoint						
Module 1^†^	0.66	0.50, 0.81	< .001	0.65	0.50, 0.81	< .001
Module 2^†^	0.62	0.48, 0.82	< .001	0.62	0.44, 0.81	< .001

Standardised estimates are reported

^†^The significant paths (*p* < .05) remain significant after the Holm method is applied for multiple testing

We conducted sensitivity tests on the direction of the midpoint paths. First, we reversed the path between midpoint home and midpoint school which resulted in a marked worsening of the goodness-of-fit (*χ*^2^ = 11.20). Secondly, we estimated the two paths simultaneously (with the removal of the covariance between the endpoints), which gave standardised estimates of 1.00 (*p* = 0.006) from parent to TA, and − 0.05 (*p* = 0.919) for TA to parent. Therefore, we fixed the path from school midpoint to home midpoint at zero.

### Model Incorporating Broader Development

General development during the course of the trial could have been a source of unobserved confounding that would have biased our estimates of generalisation paths. To assess this risk, we fitted the model of Fig. 1B which included a development factor with factor loadings all equal at 7 months (fixed at 1 for identification) and all equal at 12 months. The model likelihood improved as the variance of the development factor was reduced to zero, implying no evidence for such a factor operating for the BOSCC outcome and leaving our generalisation path estimates unchanged (Table 4).

### Moderating Factors

As general development was not found to be a significant factor in the model, moderation of generalisation was tested using the initial model. Reported in Table 5, moderation effect estimates for age, non-verbal IQ, insistence on sameness and sensory motor behaviours were all small and none even nominally significant. In each case, the models continued to fit satisfactorily. This was the case for all three paths of generalisation: (i) home midpoint to school midpoint, (ii) home midpoint to researcher endpoint, and (iii) school midpoint to researcher endpoint. The log likelihood ratio tests show there was no significant improvement in model fit when interaction terms were included.

**Table 5:** Estimates for the interaction paths exploring potential moderation of generalisation from a familiar to unfamiliar setting by non-verbal IQ, age and restricted and repetitive behaviours

	Log likelihood ratio test	Moderation	
Moderator			
Path		Coefficient for standardized moderator (95% CI)	*p*
Age			
Midpoint home to midpoint school	*χ*^2^ (1) = 0.11 *p* = 0.740	0.02 (− 0.10, 0.14)	0.772
Midpoint home to endpoint researcher	*χ*^2^ (1) = 0.52 *p* = 0.471	− 0.05 (− 0.22, 0.12)	0.593
Midpoint school to endpoint researcher	*χ*^2^ (1) = 0.17 *p* = 0.684	− 0.03 (− 0.22, 0.16)	0.747
Non-verbal IQ			
Midpoint home to midpoint school	*χ*^2^ (1) = 0.45 *p* = 0.504	− 0.05 (− 0.34, 0.23)	0.727
Midpoint home to endpoint researcher	*χ*^2^ (1) = 0.58 *p* = 0.810	− 0.03 (− 0.36, 0.30)	0.845
Midpoint school to endpoint researcher	*χ*^2^ (1) = 0.15 *p* = 0.700	− 0.07 (− 0.41, 0.28)	0.696
Repetitive behaviours—insistence on sameness			
Midpoint home to midpoint school	*χ*^2^ (1) = 1.37 *p* = 0.241	0.06 (− 0.11, 0.22)	0.507
Midpoint home to endpoint researcher	*χ*^2^ (1) = 1.57 *p* = 0.210	0.07 (− 0.12, 0.27)	0.463
Midpoint school to endpoint researcher	*χ*^2^ (1) = 2.14 *p* = 0.144	0.08 (− 0.11, 0.26)	0.410
Repetitive behaviours—sensory motor			
Midpoint home to midpoint school	*χ*^2^ (1) = 0.08 *p* = 0.772	0.01 (− 0.14, 0.16)	0.858
Midpoint home to endpoint researcher	*χ*^2^ (1) = 1.06 *p* = 0.304	0.06 (− 0.12, 0.25)	0.509
Midpoint school to endpoint researcher	*χ*^2^ (1) = 0.53 *p* = 0.465	0.04 (− 0.14, 0.23)	0.635

## Discussion

Our results build on existing evidence that autistic children do, in fact, generalise social communication skills during naturalistic development (Carruthers et al., 2020; Hong et al., 2018). Whilst intervention studies have provided evidence of generalisation by demonstrating gains in target skills within the original intervention environment, as well as a novel (generalisation) setting, the path from gains in one setting to the other is most often assumed rather than tested (see Carruthers et al., 2023; Pickles et al., 2015; Shih et al., 2021, for exceptions). The current study explicitly tests this implied path across three contexts (i.e., from the familiar settings of home and school to an unfamiliar setting), whilst controlling for the previous skill level in each context. Initial models that considered only two contexts at a time showed that children’s social communication gains with a parent between baseline and midpoint generalised to improved social communication with a researcher at endpoint. Similarly, children’s social communication gains with a teaching assistant between baseline and midpoint also generalised to improved social communication with a researcher at endpoint. In the final three-context model, the children’s generalisation of social communication from parent to researcher remained, both directly and overall, including the indirect effect via the assessment with teaching assistant at midpoint. In contrast, the generalisation effect from teaching assistant to researcher was no longer significant, and with negative point estimates. Generalisation was also shown to be significant from home midpoint to school midpoint, but not the reverse. The key generalisation paths from home and school midpoint to research endpoint did not show moderation by age, non-verbal IQ,^1^ or restricted and repetitive behaviours. In other words, these factors did not determine the extent of generalisation of social communication skills across home, school and unfamiliar contexts for the children in this sample. Our results also suggest that overall development of child behaviour across all contexts was not responsible for the pattern of results we report. We thus infer here a generalisation of a specific domain of ability (i.e., social communication skills) across context and time.

These results add new insight to the field’s growing understanding of generalisation as it applies to autistic children. Lack of a normative control group means that we cannot say with certainty that autistic children do not have difficulties with skill generalisation compared to their neurotypical peers. However, these results establish that autistic children can generalise, and should lead to reconsideration of widely reported beliefs to the contrary (Carruthers et al., 2020). Using the temporal sequences afforded by the longitudinal design, the current study also provides a novel insight into the dynamics of influence during an autistic child’s development of social communication. Generalisation of skills into the researcher BOSCC assessment was stronger from the parent BOSCC at home than from the teaching assistant BOSCC at school. When we tested the bidirectional relationship between social communication change that had occurred at home and school by midpoint, the stronger direction was from home to school. Behaviour learning accounts of generalisation hold that learning will be more readily transferred to new contexts if there is greater similarity in contingencies between the original learning context and the new one (see Swan et al., 2016 for review). More contemporary accounts are based, instead, on theories about the internalisation of procedural knowledge arising from early dyadic interaction, which can then be applied flexibly into different contexts (Carpendale & Lewis, 2004; Tomasello, 2008). Our finding that the parent-home context shows greater salience for generalisation into school and research settings supports the latter model. Home is the first occurring and enduring context in which children spend more time than other settings and involves a relationship intimacy that increases the likelihood of internalisation. There is no a priori reason why the contextual contingences of the home would be more similar to the research setting than those of the school, indeed school and research settings might be more similar.

Another factor that potentially affected generalisation from school to research settings in PACT-G may be that 63% of the children in the cohort experienced at least one change in teaching assistant at school during the study. This rate was higher than originally expected and resulted from staff turnover, staff reassignment, children changing schools, and the therapy spanning a change in school year. Whilst social communication skills do not start from scratch with each new interaction partner, for the children in the current sample who were in the midst of developing early stages of social communication, consistency (which offers routine and predictability) is likely be important (Lindsay et al., 2014).

The current study is the first to report the interplay between home and school learning environments for autistic children, though others have emphasised the importance of alignment across settings (Azad et al., 2021). For autistic children with limited verbal communication, our study suggests that while both parents and educators play a significant role in supporting learning and generalisation of skills, gains within the home environment more strongly generalise to novel environments than those from school. There are many other aspects of the educational environment, including peer relationships, group play and interaction, and the challenge of novelty and difference, that are undoubtedly essential for child social development beyond the immediate family. Further research into the dynamics between home and school learning, and their combined influence on autistic children’s social communication development, could lead to better targeted interventions.

Evidence that autistic children can and do generalise social communication skills may dictate a shift of focus, to ask what are the necessary support(s) for interventions to facilitate maximum learning and generalisation (Chang et al., 2016; Green et al., 2010). Research involving non-autistic control groups will allow further insight into the ways in which autistic generalisation may differ from ‘neurotypical’ learning patterns. For example, understanding the ways in which cognitive conceptual processes (e.g., those that underlie perceptual discrimination skills) and social-context processes (e.g., incorporating a child’s interests to facilitate peer engagement and inclusion) both interact and independently influence learning for autistic children may provide better insight into intervention strategies that promote skill generalisation.

### Strengths and Limitations

Strengths of the study include rigorous data collection, monitoring of reliability, low attrition and sample size—our analysis of 2000 separate video assessments on 248 children is substantially larger than any other study of cross-context generalisation of any skill type among autistic individuals to date. The ratings of social communication were made blind from independently recorded video, with high reliability, thus removing potential sources of confounding.

In addition to these strengths, a number of limitations are noteworthy. The primary analysis measured generalisation at the level of social communication subscale score, which is comprised of eight behaviours in Module 1 and twelve in Module 2. The resulting lack of granularity of the measure limits our understanding of the detail of generalisation. A more bounded test would have been to test generalisation of single behaviours over the three contexts. A similar distinction may be usefully drawn between downstream development and generalisation of skills (Sandbank et al., 2021). The current measurement makes it impossible to differentiate between what may have been single behaviour generalisation and a more general ‘domain’ generalisation of related skills. For instance, some children may be learning particular social communication skills (e.g., eye contact, gesture) at home and then expanding these skills into more advanced social communication skills at school (e.g., integrated social overtures); this could be more accurately described as downstream developmental effects than generalisation of specific behaviours. These factors need to be carefully considered as further tests of generalisation are designed.

The children in the PACT-G trial had high levels of autism symptoms, and largely limited language ability and low non-verbal IQ (see Table 1). These factors may limit the extent to which our findings can be generalised to the whole spectrum of autism. We administered only the nonverbal subscales of the Mullen, so were not able to control for global IQ, but the correlations between nonverbal IQ scores and language measures indicated they were strongly associated (Table S2). Furthermore, awareness of the research trial participation may have led children’s parents and teaching assistants to think more about how to develop their child’s communication, which may have influenced the learning environments of the study children in ways that may differ from children not participating in a research trial. Finally, given the null results of the PACT-G trial on the BOSCC, the current analyses were not able to benefit from the more robust causal inference possible from a successful experimental perturbation.

The BOSSC is a naturalistic, play-based measure of children’s social communication skills that can be used across time, communication partner and setting. It has strong psychometric properties and good predictive validity in relation to developmental outcomes in early intervention studies (Carruthers et al., 2021; Grzadzinski et al., 2016, 2023). Symptom-based social communication measures, such as the BOSCC, have been criticised for embodying a ‘deficit-based’ formulation of autism and prioritising neurotypical vs. autistic preferences for socialising (Timimi et al., 2019). For young children with significant developmental delay and limited communication skills, and in the context of a parent-and teacher-mediated social communication intervention trial, the BOSCC provided us with a suitable measure to test generalisation of social communication skills, despite these limitations. A recent model attempts to align traditional and neurodivergent positions within a transactional approach. This proposes that is autism an emergent entity formed through developmental processes over time between the neurodivergent brain, mind and body interacting with the social and physical environment (Green, 2022). Collaboration is needed between autistic people, their caregivers and researchers to develop approaches to early intervention and support that address the heterogeneous experiences and preferences of people on the spectrum and their families, including in the development of suitable outcome measures (Bal et al., 2018; Manzini et al., 2021).

## Conclusions

The current study builds on previous literature to add further evidence that autistic children can and do generalise social communication skills across contexts in the course of development. Using robust methods, we add novel insight into the relative contributions of different learning environments, showing that generalisation of social communication behaviours was stronger from the home setting than the school into an unfamiliar research environment when measured by an observation of naturalistic free play. Generalisation of social communication was also stronger from home to school than the reverse. We did not find any moderation of these generalisation effects by age, non-verbal IQ or level of restricted and repetitive behaviours. Future research is needed to gain a more comprehensive understanding of facilitators of generalisation in order to develop targeted strategies for interventions.

## Supplementary Information

Below is the link to the electronic supplementary material.

Supplementary file1 (DOCX 31 kb)

## Footnotes

1 Consideration of the estimated confidence intervals indicate that some caution is warranted with regard to IQ.

## Declarations

### Pre-registration

The analyses presented were preregistered (https://aspredicted.org/blind.php?x=dw43ak) with departures noted in the Supplement Materials. The analytic code necessary to reproduce the principal analyses presented are also available from https://osf.io/nxvum/. Data are available from the study PI (JG) upon reasonable request.
