# Supplementary material for: Generalisation of Social Communication Skills by Autistic Children During Play-Based Assessments Across Home, School and an Unfamiliar Research Setting
Source: J Autism Dev Disord. 2024 May 14;55(7):2203–16. doi: 10.1007/s10803-024-06370-x (PMC12167233; doi:10.1007/s10803-024-06370-x)
Supplement: Supplementary file 1 — Supplementary file1 (DOCX 31 kb) [file 10803_2024_6370_MOESM1_ESM.docx]

###### Generalisation of Social Communication Skills by Autistic Children During Play-Based Assessments Across Home, School and an Unfamiliar Research Setting

###### Supplementary Materials

**Administration of the BOSCC in PACT-G**

*Module 1*

For the researcher BOSCC, the recommended Module 1 protocol was followed of four minutes free play followed by two minutes of bubble play, and then repeated with a new set of toys. From this, four minutes of free play and one minute of bubbles was coded for each of the two segments.

For the parent and teacher BOSCCs, in order to also code the Dyadic Communication Measure of Autism (DCMA) from the same capture, the administration deviated from the recommended protocol. Each child had eight minutes of free play with the adult with the same box of toys, followed by two minutes of bubbles. From this, four minutes of play and one minute of bubbles was coded for each segment.

*Module 2*

With no recommended module 2 BOSCC administration at the start of PACT-G, the administration for the ‘verbal’ BOSCC (subsequently redeveloped into the Module 3 BOSCC), was implemented, which consists of free play and conversation. As there was some concern over whether this would be developmentally appropriate for the Module 2 children in the sample, two minutes of bubble play were added. The same toys were used as for the Module 1 children.

For the researcher BOSCC, the children had four minutes of play followed by two minutes of conversation, which was then repeated with a new set of toys, before two minutes of bubbles.

As above, in order to also code the DCMA, the parent and teacher BOSCCs had eight minutes of play with the same set of toys, four minutes of conversation, and then two minutes of bubbles. It became apparent during the trial that the conversation element was too challenging for the majority of Module 2 children and it was decided, in discussion with the BOSCC developers, that we would code the Module 2 items from four minutes of free play and one minute of bubbles in each segment.

**BOSCC Social Communication items – Module 1**

1 Eye contact

2 Facial expression

3 Gestures/show

4 Vocalisation directed to others

5 Integration of vocal and non-vocal modes of communication

6 Frequency and function of social overtures

7 Frequency and quality of responses

8 Engagement in play activities/interaction

**BOSCC Social Communication items – Module 2**

1 Eye contact

2 Facial expression

3 Gestures/show

4 Vocalisation directed to others

5 Integration of vocal and non-vocal modes of communication

6 Frequency of social overtures

7 Quality of social overtures

8 Frequency of social responses

9 Quality of social responses

10 Verbal exchanges

11 Offering information

12 Engagement in play activities/interaction

**Changes to analysis compared to preregistration**

- The analysis on the RRB subscale was not run as the naturalistic interaction, brevity and setting of the assessment resulting in infrequent RRBs that were insufficient for a reliable analysis of the required complexity.
- The timepoints were identified as 6 months for parent midpoint and 11 months for endpoint in the preregistration. In this paper we have referred to these as 7 months for midpoint for both parent and teacher and 12 months for endpoint to be consistent with other PACT-G publications.
- An additional analysis was conducted to explore whether the addition of a latent factor representing general child development over the 12-month period changed the pattern of results.
- The additional sensitivity analysis to test for difference in the main non-standardized generalization path coefficient from midpoint parent to teacher failed to converge.

**Analysis Code**

TITLE: BOSCC cross context generalisation - Social Communication without moderation.

DATA: FILE = statadata.dat ;

VARIABLE: Names are

module t_0 t_8 t_11 r_0 r_11 p_0 p_6 p_11 agegrp gender s2 s3 trt

r_sc0 r_sc11 p_sc0 p_sc6 p_sc11 t_sc0 t_sc8 t_sc11 tch8m tch11m iq

RBQ_sm RBQ_is age0 id;

USEVARIABLES module agegrp gender s2 s3 trt r_sc0 r_sc11 p_sc0

p_sc6 p_sc11 t_sc0 t_sc8 t_sc11 tch8m tch11m;

GROUP IS module (1 = mod1, 2 = mod2);

Missing are all (-9999) ;

ANALYSIS: estimator is ML;

MODEL:

!HOME LATENT

P0 BY p_sc0@1;

P6 BY p_sc6@1;

P11 BY p_sc11@1;

[P0];

[P6];

[P11];

!SCHOOL LATENT

T0 BY t_sc0@1;

T8 BY t_sc8@1;

T11 BY t_sc11@1;

[T0];

[T8];

[T11];

!Home context autoregressive and stratifiers

P0 on agegrp gender s2 s3;

P6 ON P0 ;

P11 ON P6;

!Home context treatment paths

P6 ON trt (h);

P11 ON trt (hh);

!School context autoregressive and stratifiers

T0 on agegrp gender s2 s3;

T8 ON T0 ;

T11 ON T8;

!School context treatment paths

T8 ON trt (z);

T11 ON trt (zz);

!Change in teacher

T8 ON tch8m (g);

T11 ON tch11m (g);

!Researcher context autoregressive and stratifiers

r_sc11 ON r_sc0 (c);

r_sc0 ON agegrp gender s2 s3;

!Generalisation paths

P6 ON T8@0;

T8 ON P6 (y);

r_sc11 ON T8 (a);

r_sc11 ON P6 (b);

!Zero trt to baseline

r_sc0 ON trt@0;

P0 ON trt@0;

T0 ON trt@0;

!Baseline correlations

r_sc0 WITH P0;

r_sc0 WITH T0;

P0 WITH T0;

!Endpoint correlations

r_sc11 WITH P11;

r_sc11 WITH T11;

P11 WITH T11;

!Constrain residual variance of observed variables to be equal across time and module

p_sc0 p_sc6 p_sc11 (j);

t_sc0 t_sc8 t_sc11 (o);

MODEL INDIRECT:

r_sc11 ind P6;

MODEL CONSTRAINT: 0 < o;

OUTPUT: stand cinterval (bootstrap);

**Table S1** Results of Wald tests used in testing paths and constraints for model.

| **Path** | **Constraint** | **Test** | **Test result** | **Conclusion** |
| --- | --- | --- | --- | --- |
| Autoregressive paths between latent factors  Home and School paths | Equality constraints across module | LR test | χ^2^ (4) = 23.7  p < .001 | Leave unconstrained |
| Autoregressive path between researcher contexts | Equality constraints across module | LR test | χ^2^ (1) = 0.61  p = 0.436 | Constrain equal |
| Paths between school midpoint/endpoint and intervention group | Equality constraints across module | Wald test  *Null hypothesis: the path is equal across Modules* | Group -> School midpoint  χ^2^ (1) = 0.02  p = 0.879  Group -> School endpoint  χ^2^ (1) = 0.134  p = 0.714 | Constrain both paths equal across Module |
| Paths between home midpoint/endpoint and intervention group | Equality constraints across module | Wald test  *Null hypothesis: the path is equal across Modules* | Group -> Home midpoint  χ^2^ (1) = 0.643  p = 0.423  Group -> Home endpoint  χ^2^ (1) = 2.78  p = 0.096 | Constrain both paths equal across Module |
| Paths between school midpoint, endpoint and change in teaching assistant | Equality constraints across module  &  Equality of path from change in TA to midpoint and change in TA to endpoint | Wald test  *Null hypotheses: the path is equal across Modules; the paths are equal* | Change in TA at midpoint:  χ^2^ (1) = 1.97  p = 0.161  Change in TA at endpoint:  χ^2^ (1) = 0.034  p = 0.855  Equality of the two paths:  χ^2^ (1) = 1.15  p = 0.283 | Constrain both paths equal across Module  &  Constrain both paths equal to each other |
| School midpoint -> research endpoint | Equality constraints across module | Wald test  *Null hypothesis: the path is equal across Modules* | χ^2^ (1) = 0.313  p = 0.576 | Constrain equal across Module |
| Home midpoint -> research endpoint | Equality constraints across module | Wald  *Null hypothesis: the path is equal across Modules* | χ^2^ (1) = 0.263  p = 0.608 | Constrain equal across Module |
| Home midpoint -> school midpoint | Equality constraints across module |  | Final model not empirically identified without this constraint | Constrain equal across Module |
| Correlations between endpoints | To fix to zero or leave unconstrained | LR test | χ^2^(6) = 11.356  p = 0.088 | Although the LR test suggests fixing endpoint-correlations to zero would improve model fit, allowing the correlation reduces bias as it allows for correlation induced by shared confounding. Leaving endpoint correlations unconstrained gave a solution with positive-definite latent variable covariance matrix. |
| Residual variances for observed home and school variables | Equality constraints across module & timepoint |  | Final model not empirically identified without this constraint | Constrain equal across Module and timepoint |

*Note.*

LR test = Likelihood Ratio test, TA = teaching assistant. With the LR test, if this difference is statistically significant, then the less restrictive model (the one with more variables) is said to fit the data significantly better than the more restrictive model. With the Wald test, a significant result rejects the null set at the time of the test, e.g. that the path is equal across the Modules.

**Table S2** Baseline correlations between measures of key child characteristics.

|  | Mullen Visual Reception | MCDI says and understood | Receptive One Word | Expressive One Word | SDQ disruptive | RBQ Sensory Motor | RBQ Insistence on Sameness | ADOS module  (Spearman) |
| --- | --- | --- | --- | --- | --- | --- | --- | --- |
| Mullen Visual Reception |  |  |  |  |  |  |  |  |
| MCDI says and understood | 0.60** |  |  |  |  |  |  |  |
| Receptive One Word | 0.74** | 0.76** |  |  |  |  |  |  |
| Expressive One Word | 0.72** | 0.84** | 0.88** |  |  |  |  |  |
| SDQ disruptive | 0.01 | 0.05 | 0.05 | 0.07 |  |  |  |  |
| RBQ Sensory Motor | -0.04 | -0.18* | -0.14* | -0.13 | 0.25** |  |  |  |
| RBQ Insistence on Sameness | 0.23** | 0.18* | 0.15* | 0.18* | 0.26** | 0.51** |  |  |
| ADOS module (Spearman) | 0.51** | 0.64** | 0.57** | 0.65** | 0.14 | -0.06 | 0.25** |  |
| ADOS CSS  (Spearman) | -.21* | 0.15* | -0.12 | 0.008 | 0.06 | -0.03 | -0.12 | 0.24** |

*Note.*

These correlations were used to explore potential additional moderators. Mullen Visual Reception, RBQ Sensory Motor and RBQ Insistence on Sameness had been selected a priori. ADOS module is also already included in the model. **<.001 *<.05
